# Comparison of strain parameters in dyssynchronous heart failure between speckle tracking echocardiography vendor systems

**DOI:** 10.1186/s12947-017-0116-5

**Published:** 2017-10-18

**Authors:** Wouter M. van Everdingen, Alexander H. Maass, Kevin Vernooy, Mathias Meine, Cornelis P. Allaart, Frederik J. De Lange, Arco J. Teske, Bastiaan Geelhoed, Michiel Rienstra, Isabelle C. Van Gelder, Marc A. Vos, Maarten J. Cramer

**Affiliations:** 1 Department of Cardiology, University Medical Centre Utrecht, P.O. Box 855500, 3508 GA Utrecht, The Netherlands; 2 Department of Cardiology, Thoraxcenter, University of Groningen, University Medical Centre Groningen, Groningen, The Netherlands; 3 Department of Cardiology, Maastricht University Medical Centre, Maastricht, The Netherlands; 4 Department of Cardiology, VU University Medical Centre, Amsterdam, The Netherlands; 5 Department of Cardiology, Academic Medical Centre, Amsterdam, The Netherlands; 6 Department of Medical Physiology, University of Utrecht, Utrecht, The Netherlands

**Keywords:** Speckle tracking echocardiography, Cardiac resynchronization therapy, Strain, Dyssynchrony, Heart failure, Vendor comparison, Response

## Abstract

**Background:**

Although mechanical dyssynchrony parameters derived by speckle tracking echocardiography (STE) may predict response to cardiac resynchronization therapy (CRT), comparability of parameters derived with different STE vendors is unknown.

**Methods:**

In the MARC study, echocardiographic images of heart failure patients obtained before CRT implantation were prospectively analysed with vendor specific STE software (GE EchoPac and Philips QLAB) and vendor-independent software (TomTec 2DCPA). Response was defined as change in left ventricular (LV) end-systolic volume between examination before and six-months after CRT implantation. Basic longitudinal strain and mechanical dyssynchrony parameters (septal to lateral wall delay (SL-delay), septal systolic rebound stretch (SRSsept), and systolic stretch index (SSI)) were obtained from either separate septal and lateral walls, or total LV apical four chamber. Septal strain patterns were categorized in three types. The coefficient of variation and intra-class correlation coefficient (ICC) were analysed. Dyssynchrony parameters were associated with CRT response using univariate regression analysis and C-statistics.

**Results:**

Two-hundred eleven patients were analysed. GE-cohort (*n* = 123): age 68 years (interquartile range (IQR): 61–73), 67% male, QRS-duration 177 ms (IQR: 160–192), LV ejection fraction: 26 ± 7%. Philips-cohort (*n* = 88): age 67 years (IQR: 59–74), 60% male, QRS-duration: 179 ms (IQR: 166–193), LV ejection fraction: 27 ± 8. LV derived peak strain was comparable in the GE- (GE: -7.3 ± 3.1%, TomTec: −6.4 ± 2.8%, ICC: 0.723) and Philips-cohort (Philips: −7.7 ± 2.7%, TomTec: −7.7 ± 3.3%, ICC: 0.749). SL-delay showed low ICC values (GE vs. TomTec: 0.078 and Philips vs. TomTec: 0.025). ICC’s of SRSsept and SSI were higher but only weak (GE vs. TomTec: SRSsept: 0.470, SSI: 0.467) (Philips vs. QLAB: SRSsept: 0.419, SSI: 0.421). Comparability of septal strain patterns was low (Cohen’s kappa, GE vs. TomTec: 0.221 and Philips vs. TomTec: 0.279). Septal strain patterns, SRSsept and SSI were associated with changes in LV end-systolic volume for all vendors. SRSsept and SSI had relative varying C-statistic values (range: 0.530–0.705) and different cut-off values between vendors.

**Conclusions:**

Although global longitudinal strain analysis showed fair comparability, assessment of dyssynchrony parameters was vendor specific and not applicable outside the context of the implemented platform. While the standardization taskforce took an important step for global peak strain, further standardization of STE is still warranted.

**Electronic supplementary material:**

The online version of this article (10.1186/s12947-017-0116-5) contains supplementary material, which is available to authorized users.

## Background

Speckle tracking echocardiography (STE) is used to assess myocardial deformation and strain in research setting as well as in clinical practice [1, 2]. The use of STE in cardiac resynchronization therapy (CRT) has received increasing interest the past years, with respect to multiple aspects: optimization of left ventricular (LV) lead positioning, myocardial viability, optimization of CRT device configuration, determining mechanical dyssynchrony, and predicting volumetric response and outcome [3–7]. Response prediction is an important aspect of clinical decision making, since 20–50% of patients are still non-responders to CRT despite meeting internationally acknowledged selection criteria [8]. Prediction of volumetric response and outcome to CRT has been approached using several STE derived parameters for mechanical dyssynchrony [3, 7, 9, 10]. Publications on these parameters mainly use STE software of General Electric EchoPac (Chicago, Illinois, United States) [9, 11, 12]. However, several other commercially available vendor dependent and independent software platforms have been developed for STE [9, 10, 13]. Between these platforms, differences in derived results are known, complicating the interpretation of specific study results and restricting their use in clinical practice [14, 15]. A taskforce of the European Association of Cardiovascular Imaging and American Society of Echocardiography (EACVI/ASE) was appointed to standardize longitudinal strain results and specifically global values [16]. However, inter vendor comparability of results obtained in patients with LV dyssynchrony is unknown. It was the aim of this study to compare strain parameters and more specifically dyssynchrony parameters derived from longitudinal strain analysis of different vendors of STE software, implemented specifically in CRT patients, as well as the association of derived dyssynchrony parameters with volumetric response to CRT. STE software of two commonly used vendors was used (i.e. GE EchoPac and Philips QLAB (Philips Medical Systems, Best, The Netherlands)), and the vendor-independent system of TomTec 2DCPA (TomTec Imaging Systems GmbH, Unterschleissheim, Germany). The hypothesis of this study is that vendors may have good agreement on global parameters and timing indices in patients eligible for CRT, while agreement on more detailed parameters and dyssynchrony parameters may be poor.

## Methods

### Study design

The Markers and Response to CRT (MARC) study was designed to investigate predictors for response on CRT, including several echocardiographic parameters [17]. The study was initiated and coordinated by the six centres within the framework of the Centre for Translational Molecular Medicine (CTMM), project COHFAR (grant 01C-203), and additionally supported by Medtronic (Fridley, Minnesota, USA). Study monitoring was done by Medtronic, data management and validation by the investigators (MR, BG) in collaboration with Medtronic. The study was approved by the institutional review boards of all participating centres. All patients gave written informed consent. The trial was registered at clinicaltrials.gov: NCT01519908.

### Study participants

Two hundred forty patients eligible for CRT according to the most recent international guidelines were included in the MARC study [18, 19]. In short, MARC study inclusion criteria were: sinus rhythm and optimal pharmacological heart failure therapy, QRS-duration ≥130 ms in patients with left bundle branch block (LBBB) and QRS-duration of ≥150 ms in non-LBBB patients with NYHA class II and QRS-duration ≥120 ms in LBBB patients with NYHA class III. Exclusion criteria were severe renal insufficiency, an upgrade from a bradycardia pacemaker or CRT-P to CRT-D, permanent atrial fibrillation, flutter or tachycardia, right bundle branch block, and permanent 2nd or 3rd degree atrioventricular block. Before and 6 months after CRT implantation, data were recorded at the outpatient department, including electrocardiographic and echocardiographic examination. Patients were excluded for this sub-analysis if frame rate of the apical four chamber (4CH) view was below 35 Hz, in case of irregular heart rhythm, unanalysable images due to technical errors or if image quality was very poor.

#### Echocardiographic examination

Echocardiographic examinations were performed by participating centres and analysed at the echocardiographic core lab situated in the UMC Utrecht (Utrecht, the Netherlands). Echocardiographic examinations made in this study were performed on either GE Vivid7, GE Vivid9, or Philips iE33 ultrasound machines. Standard images included a 4CH view, zoomed and focused on the LV. Of these images both image quality and frame rate were optimized for offline analysis. Analysis of apical rocking and interventricular mechanical delay (IVMD) are described in earlier work [17]. Pulsed-wave Doppler images of the LV outflow tract were obtained for definition of aortic valve closure time. QRS-onset and aortic valve closure time were used to define systole.

### Volumetric response

LV ejection fraction, LV end-diastolic and end-systolic (LVESV) volumes were measured by biplane Simpson’s method [20]. Volumetric response to CRT was defined as the percentage of change in LVESV between echocardiographic examination before and 6 months after CRT implantation. Patients were classified as responder in case of ≥15% reduction in LVESV.

### Speckle tracking echocardiography

Echocardiographic 4CH images were subjected to offline speckle tracking analysis (WE, MC). The optimal images for speckle tracking were selected and used for the vendor dependent and independent platform (Additional file 1: Figure S1). All images were scored for quality (poor, average, or high) by two experienced observers. Image quality was categorized as high if the total LV myocardium was visible during the entire cardiac cycle, average if one or two segments were not clearly visible and poor in all other cases. Images were exported to vendor specific software (GE EchoPac 11.3 and Philips QLAB 10.0) in standard formats and exported as DICOM-files for vendor independent software (TomTec 2D Cardiac Performance Analysis (2DCPA) version 1.2.1.2). Speckle tracking was performed with standard settings for all vendors. For each platform, a region of interest (ROI) was placed by user defined markers to incorporate the entire myocardial wall. Repeat adjustments of the ROI were done if tracking quality was insufficient. The myocardial wall was separated into six segments by all platforms (i.e. basal and mid inferoseptal, apical septal, apical lateral and basal and mid anterolateral). Philips QLAB analyses an additional true apical segment (i.e. 17 segment model of the AHA), which was excluded for the septal and lateral wall strain curves, as it was part of both walls [21]. Segments were also excluded if adequate tracking was not achievable. The basal inferoseptal, mid inferoseptal and apical septal segment were averaged into a global septal wall strain curve. The apical lateral, basal anterolateral and mid anterolateral segment were averaged into a global lateral wall strain curve. Results from a single wall were excluded if tracking of more than one segment was unachievable. The entire myocardial wall was used for both Philips and GE analysis. TomTec analysis resulted in separate datasets for the endocardial and epicardial border. The epicardial border at the apical and mid ventricular lateral wall was often outside the echocardiographic window, and was therefore excluded in TomTec analysis. This was done even though differences between endo- and epicardial layers are known [22]. The marker for reference length (L_0_) was placed at onset of QRS-complex for GE derived images, both for GE EchoPac and TomTec 2DCPA. L_0_ of Philips derived images could not be altered in QLAB and was automatically placed in the QRS-complex. L_0_ was manually placed at a similar position for Philips derived images analysed with TomTec 2DCPA. Therefore, both direct comparisons (GE vs. TomTec and Philips vs. TomTec) had similar L_0_ positions.

### Offline analysis

Results of speckle tracking analysis were stored and exported for offline analysis with Matlab 2014b (Mathworks, Natick, MA, USA). Author written Matlab scripts allowed for input of valve closure times and semi-automatic calculation of strain parameters. Results of strain parameters were based on global strain curves. Global strain curves were averages of the segments representing the global LV or the separate septal or lateral wall.

#### Parameters

##### Basic strain parameters

**Fig. 1:**
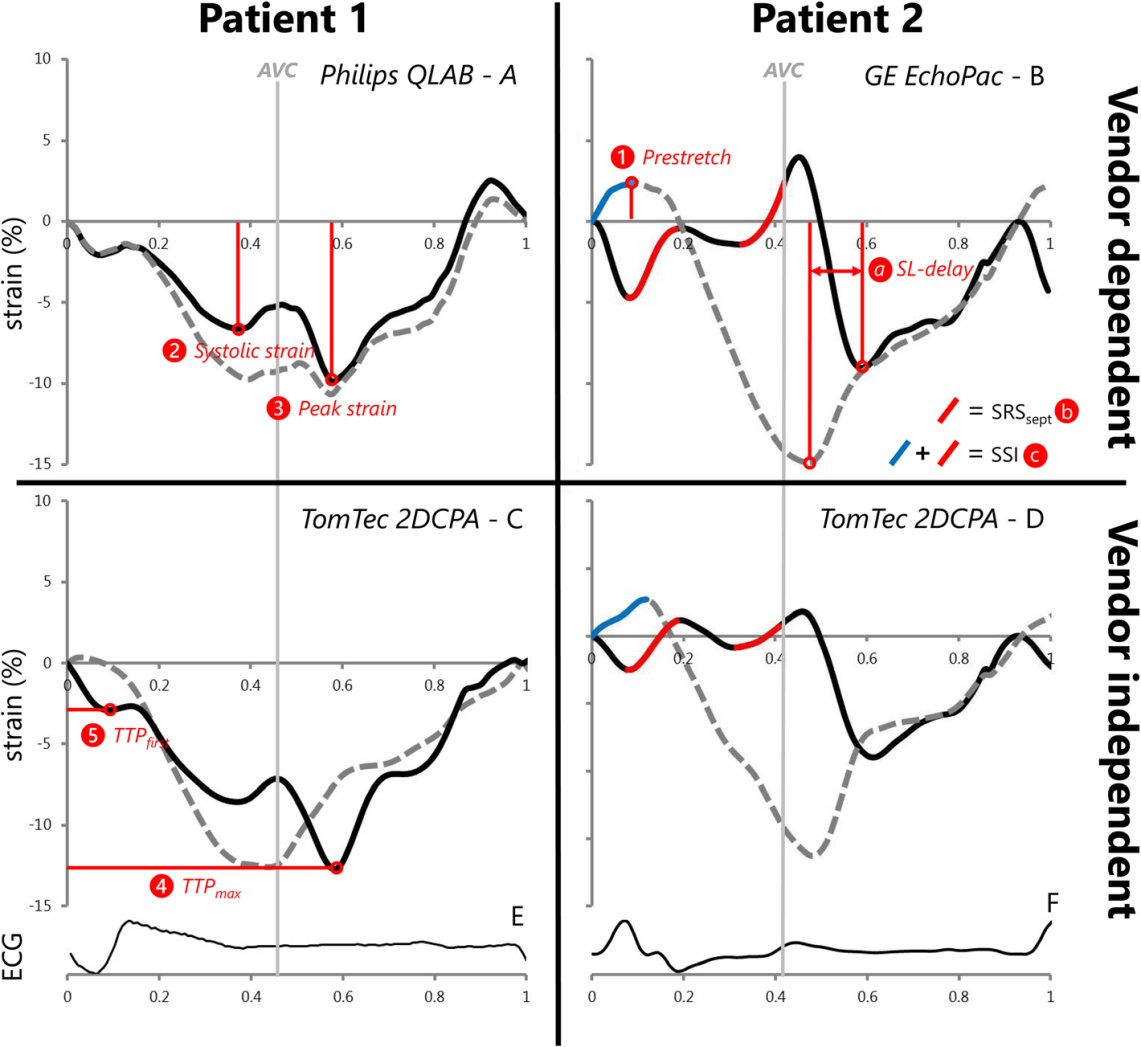
Examples of strain curves of the septal (solid black curve) and lateral wall (dashed grey curve) derived with vendor dependent and independent speckle tracking echocardiography software of two patients. Aortic valve closure (AVC) is marked with a thin solid grey line. Philips was used for echocardiographic examination in patient 1 (panel A & C), and GE was used for patient 2 (panel B & D). Corresponding ECGs are shown below (panel E & F). SRSsept is marked red and lateral wall pre-stretch is marked blue in patient 2. AVC: aortic valve closure, MVC: mitral valve closure, SL-delay: septal to lateral wall delay, SRSsept: septal systolic rebound stretch, SSI: systolic stretch index, TTP_first_: time to first peak shortening, TTP_max_: time to maximal peak shortening

Five basic strain parameters were obtained for global LV, septal wall and lateral wall strain curves. 1) Pre-stretch was defined as maximal positive peak strain, occurring after QRS onset and before shortening (Fig. 1). 2) Peak strain was the maximal negative peak strain during the entire cardiac cycle. 3) Systolic strain was the maximal negative strain during systole. 4) Time to maximal peak (TTP_max_) was the time difference from L_0_ to most negative peak strain. 5) Time to first peak (TTP_first_) was the time difference from L_0_ to first negative peak.

##### Dyssynchrony parameters

**Fig. 2:**
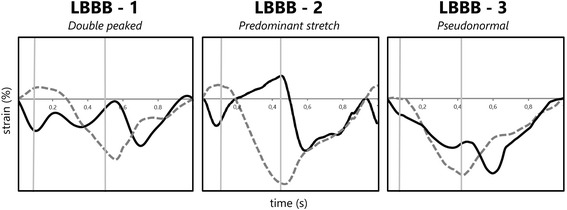
Examples of septal strain pattern types. Septal strain patterns are categorized in three types: LBBB-1: double peak rebound stretch, LBBB-2: predominant stretch and LBBB-3: pseudo normal shortening, according to Leenders et al. [25] The septal strain curve is displayed as a solid black line, while the lateral wall strain curve is displayed as a dashed grey line. LBBB: left bundle branch block

Four dyssynchrony parameters were compared. a) Septal to lateral wall delay (SL-delay) was calculated as the difference in TTP_max_ of the septal and lateral walls. b) Septal systolic rebound stretch (SRSsept) was defined as the cumulative amount of stretch after initial shortening of the septum, occurring during systole (Fig. 1) [3]. c) Systolic stretch index (SSI) was defined as the sum of SRSsept and lateral wall pre-stretch [9]. d) Septal strain curves were categorized in three LBBB pattern types, determined by their shape, based on earlier work of our group [23]. LBBB-1: double-peaked systolic stretch, LBBB-2: early pre-ejection shortening peak followed by prominent systolic stretching and LBBB-3: pseudo normal shortening with a late-systolic shortening peak followed by less pronounced end-systolic stretch (Fig. 2).

##### Cross-correlation

The similarity of strain curves between vendor dependent and independent software was analysed by cross correlation of strain signals obtained from the same patient and image. Strain data of the vendor dependent analysis was interpolated and plotted on the horizontal axis, while data of the vendor independent analysis was plotted on the vertical axis. Least squares fitting (y = a*x) of this data was used to calculate the coefficient of determination (R^2^). Strain data of the vendor dependent analysis was shifted by steps of 1 ms and R^2^ was calculated for each step. After a total shift of 100 ms, the highest value was used as the optimal correlation coefficient.

#### Intra-observer agreement

Categorization of septal strain curves of all patients were analysed a second time with vendor-specific and vendor independent software for intra-observer agreement. There was an interval of at least 20 weeks between the data-analyses.

#### Statistical analysis

Statistical analysis was performed (BG and MR) using R version 3.2.4 (The R foundation for Statistical Computing), SAS software version 9.4 (SAS Institute, Cary, NC, USA) and the R-packages psych version 1.5.8 (for calculation of Cohen’s kappa coefficients, ICCs and their associated *p*-values). Comparison of subgroups on baseline characteristics and strain parameters of GE and Philips was performed using a student t-test or Wilcoxon test, dependent on normality of data. Categorical data was compared using a Fisher exact test or Chi-Square tests if more than two categories were present. To compare vendor dependent to vendor independent data, strain parameters were compared by a paired t-test or Wilcoxon test, dependent on normality of data. The coefficient of variation (COV), intra-class correlation coefficient (ICC), and Bland-Altman plots were also used for comparison between vendors. For Bland-Altman plots, the mean, standard deviation and 95% confidence interval (i.e. limits of agreement) were calculated. Cross-correlation results were compared using a pairwise t-test with Bonferroni correction. Agreement of LBBB pattern categorization was assessed using Cohen’s kappa coefficient. ICC and Cohen’s kappa results were classified as follows; ≥0.75: excellent, 0.60–0.74: good, 0.40–0.59: weak, and <0.40: poor. Univariate regression analysis with change in LVESV as a continuous variable was used to test dyssynchrony parameters as predictors for response to CRT. The C-statistic and cut-off value were calculated for each dyssynchrony parameter, with volumetric response (LVESV reduction ≥15%) as a dichotomous parameter. A *p*-value <0.05 was considered significant for all tests.

## Results

### Study population

Two-hundred-eleven of 240 MARC study patients were included in this sub-analysis, 123 in the GE-cohort and 88 in the Philips-cohort. Nineteen patients were excluded for GE analysis, of which five were excluded from the main study, two had irregular heart rhythm, four had a frame rate below 35 Hz, four had overall low image quality and two had only one analysable segment for the lateral wall. Ten patients were excluded for Philips analysis, of which four were already excluded from the main study, five were stored in a datafile not analysable for STE and one had a frame rate below 35 Hz. There were no significant differences in baseline characteristics between cohorts (Table 1), except for frame rate. Frame rate was higher in the GE-cohort (61 ± 12 Hz) compared to the (Philips-cohort 55 ± 7 Hz, *p* < 0.001). LV end-diastolic and end-systolic volumes tended to be lower in the Philips-cohort compared to the GE-cohort. LV ejection fraction was comparable, as were conventional electrical dyssynchrony (i.e. QRS duration and morphology) and mechanical dyssynchrony parameters (i.e. IVMD, apical rocking and septal flash). IVMD was above the cut-off value of 40 ms in both groups, septal flash was seen in approximately half of all patients, while apical rocking was observed in around 60% of patients. CRT response rate was non-significantly different in the two cohorts (GE-cohort: 59% vs. Philips-cohort: 65%), with non-significantly differences in ESV reduction (GE-cohort: 20 ± 23% vs. Philips-cohort: 25 ± 26, *p* = 0.208).

**Table 1:** Baseline characteristics

	GE-cohort (*n* = 123)	Philips-cohort (*n* = 88)	*p*-value
Age (years)	68.3 (61.3–73.4)	67.2 (59.0–73.9)	0.450
Gender (n, % male)	82 (66.7%)	53 (60.2%)	0.384
BMI (kg/m^2^)	26.5 (23.8–29.6)	26.2 (23.6–29.3)	0.813
NYHA Class (n, %)			
I	1 (0.8%)	0 (0.0%)	0.869
II	77 (62.6%)	53 (60.2%)	
III	45 (36.6%)	35 (39.8%)	
QRS duration (ms)	177 (160–192)	179 (166–193)	0.293
QRS morphology (n, %)			
LBBB	68 (56.7%)	55 (64.7%)	0.311
IVCD	52 (43.3%)	30 (35.3%)	
LVEDV (ml)	183.3 (148.8–247.7)	168.0 (132.0–211.8)	0.051
LVESV (ml)	135.3 (100.7–194.7)	130.3 (92.8–167.3)	0.087
LVEF (%)	25.6 ± 7.3	26.5 ± 7.9	0.406
LVEDD (cm)	6.3 ± 0.8	6.2 ± 0.8	0.591
IVMD (ms)	47.1 ± 28.8	46.3 ± 30.2	0.855
Apical rocking (n, %)	71 (58.2%)	56 (63.6%)	0.476
Septal flash (n, %)	56 (47.5%)	42 (48.8%)	0.888
Frame rate (Hz)	61 ± 12	55 ± 7	<.001
Image quality (n, %)			
Poor	31 (25.2%)	8 (9.1%)	0.011
Average	54 (43.9%)	50 (56.8%)	
High	38 (31.0%)	30 (34.1%)	
ESV reduction	20.4 ± 22.9	24.9 ± 25.7	0.208
Responders (n, %)	65 (58.6%)	54 (65.1%)	0.375

Standard deviations are given with ± symbol, for non-normal distributed data, the median is given with the interquartile range between brackets

*BMI* body mass index, *NYHA* New York Heart Association, *LBBB* left bundle branch block, *LV* left ventricular, *LVEDV* LV end-diastolic volume, *LVESV* LV end-systolic volume, *LVEF* LV ejection fraction, *LVEDD* LV end-diastolic diameter, *IVCD* intra-ventricular conduction delay, *IVMD* interventricular mechanical delay

### GE echocardiographic images

#### GE basic strain parameters

**Fig. 3:**
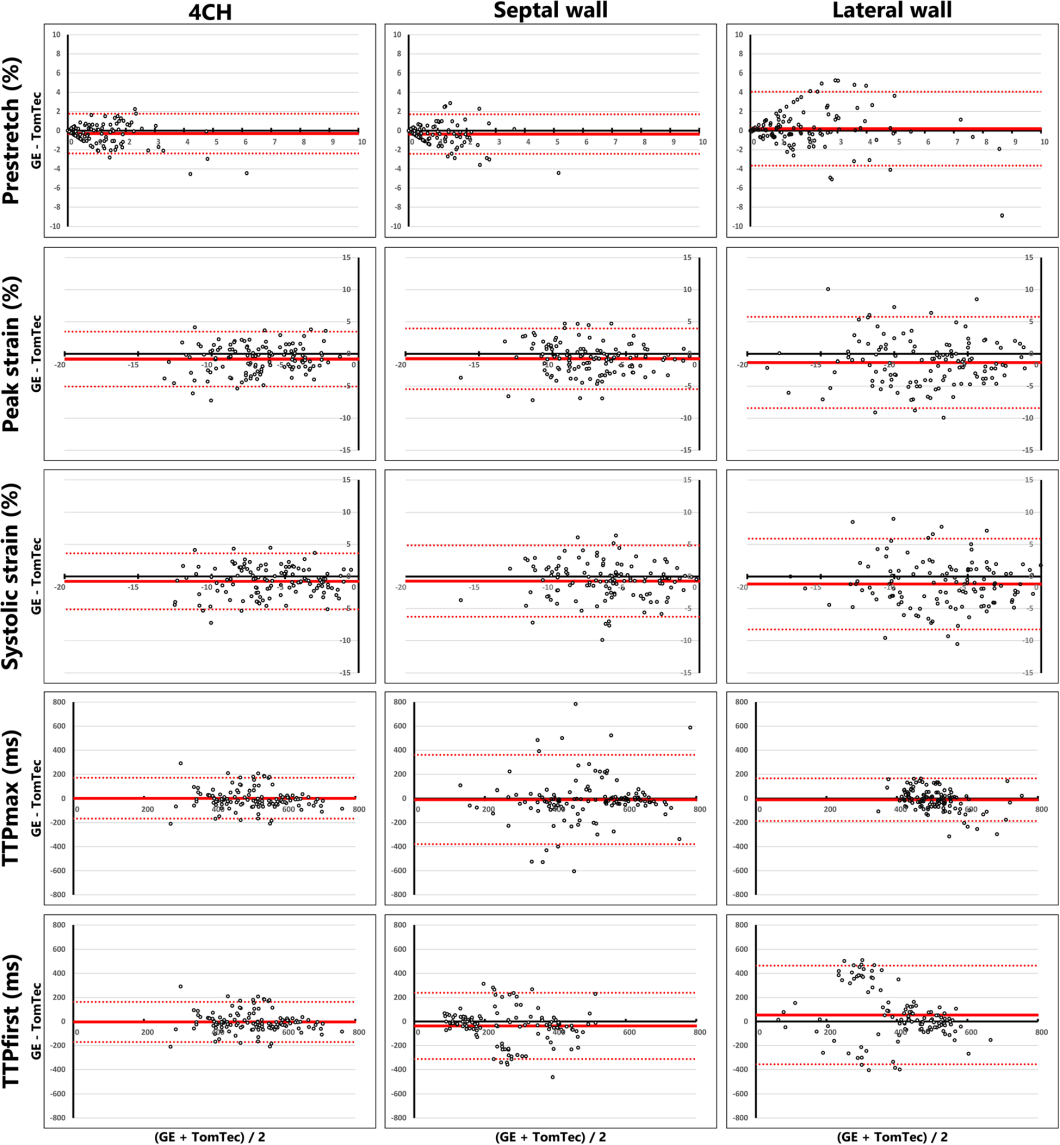
Bland-Altman plots of all strain parameters, comparing GE Echopac and TomTec 2DCPA derived results. Each column represents either results obtained from the total LV or from the separate septal or lateral wall. On each x-axis the average result of the two techniques is given per patient, while on the y-axis the difference is given. 4CH: apical four chamber view, GE: General Electric, TTP_first_: time to first peak, TTP_max_: time to maximal peak

Comparison of strain results obtained with vendor dependent and independent STE software resulted in a good to excellent ICC for peak strain and systolic strain for global LV and septal wall (Table 2). COV was relatively low, as was the mean difference in Bland-Altman plots (Fig. 3). Nevertheless, the standard deviations of the Bland-Altman plots were relatively large, ranging from 2.2 to 2.8%. The ICC of peak and systolic strain of the lateral wall were weak (0.595 and 0.565 respectively), with an even larger standard deviation in Bland-Altman plots (3.6 and 3.7%, respectively). The ICC of TTP_first_ and TTP_max_ of both walls and the global LV were poor to weak, with relatively large COV and large standard deviations in Bland-Altman plots.

**Table 2:** Strain parameters derived from GE echocardiographic images

	GE EchoPac (*n* = 123)	TomTec 2DCPA (*n* = 123)	COV	ICC (*p*-value)	Bland-Altman (mean diff ±SD)
LV					
1) Pre-stretch (%)	0.4 (0.0–1.4)	0.7 (0.1–1.7)	1.218	0.631 (<0.001)	−0.3 ± 1.0
2) Peak strain (%)	−7.3 ± 3.1	−6.4 ± 2.8	−0.424	0.723 (<0.001)	−0.8 ± 2.2
3) Systolic strain (%)	−6.4 ± 3.2	−5.6 ± 3.2	−0.504	0.752 (<0.001)	−0.8 ± 2.2
4) TTP_max_ (ms)	511 (426–587)	488 (429–593)	0.201	0.676 (<0.001)	−2 ± 86
5) TTP_first_ (ms)	400 (158–458)	421 (316–471)	0.480	0.195 (0.015)	−34 ± 205
Septum					
1) Pre-stretch (%)	0.3 (0.0–1.0)	0.7 (0.0–1.5)	1.337	0.470 (<0.001)	−0.4 ± 1.1
2) Peak strain (%)	−8.0 ± 3.1	−7.2 ± 3.2	−0.392	0.707 (<0.001)	−0.8 ± 2.4
3) Systolic strain (%)	−6.7 ± 3.5	−6.0 ± 3.5	−0.517	0.667 (<0.001)	−0.7 ± 2.8
4) TTP_max_ (ms)	531 (378–626)	520 (414–606)	0.336	0.261 (0.002)	−9 ± 189
5) TTP_first_ (ms)	208 (135–376)	311 (151–420)	0.521	0.486 (<0.001)	−39 ± 142
Lateral wall					
1) Pre-stretch (%)	1.6 (0.5–3.1)	1.2 (0.2–2.4)	0.951	0.524 (<0.001)	0.3 ± 1.9
2) Peak strain (%)	−8.5 (−11.4- -5.8)	−6.5 (−10.2- -4.3)	−0.462	0.595 (<0.001)	−1.4 ± 3.6
3) Systolic strain (%)	−6.6 (−10.8- -4.2)	−5.5 (−9.0- -3.0)	−0.532	0.565 (<0.001)	−1.2 ± 3.7
4) TTP_max_ (ms)	500 (456–541)	514 (445–556)	0.149	0.444 (<0.001)	−7 ± 101
5) TTP_first_ (ms)	475 (419–520)	431 (300–522)	0.302	0.136 (0.066)	47 ± 206
Dyssynchrony					
a) SL-delay (ms)	25 (−132–110)	−13 (−121–101)	−14.440	0.078 (0.194)	−2 ± 226
b) SRSsept (%)	1.7 (0.8–3.4)	1.1 (0.1–1.9)	0.937	0.470 (<0.001)	1.0 ± 2.0
c) SSI (%)	3.8 (2.1–5.9)	2.6 (1.3–3.8)	0.720	0.467 (<0.001)	1.3 ± 3.0
d) LBBB type (n, %)					
LBBB-1	46 (37.4%)	36 (29.3%)			
LBBB-2	17 (13.8%)	10 (8.1%)			
LBBB-3	60 (48.8%)	77 (62.6%)			

Means and standard deviations are given with ± symbol. For non-normal distributed data, the median is given with the interquartile range between brackets

*COV* coefficient of variation, *ICC* intra-class correlation coefficient, *diff* difference, *SD* standard deviation, *LV* strain derived from global LV in apical four chamber view, *TTP*_*max*_ time to maximal peak shortening, *TTP*_*first*_ time to first peak shortening, *SL*-*delay* time delay between septal and lateral peak shortening, *SSI* systolic stretch index, *SRSsept* septal systolic rebound stretch, *LBBB type* type of LBBB strain patterns, based on definition by Leenders et al. [23]

#### GE dyssynchrony parameters

**Fig. 4:**
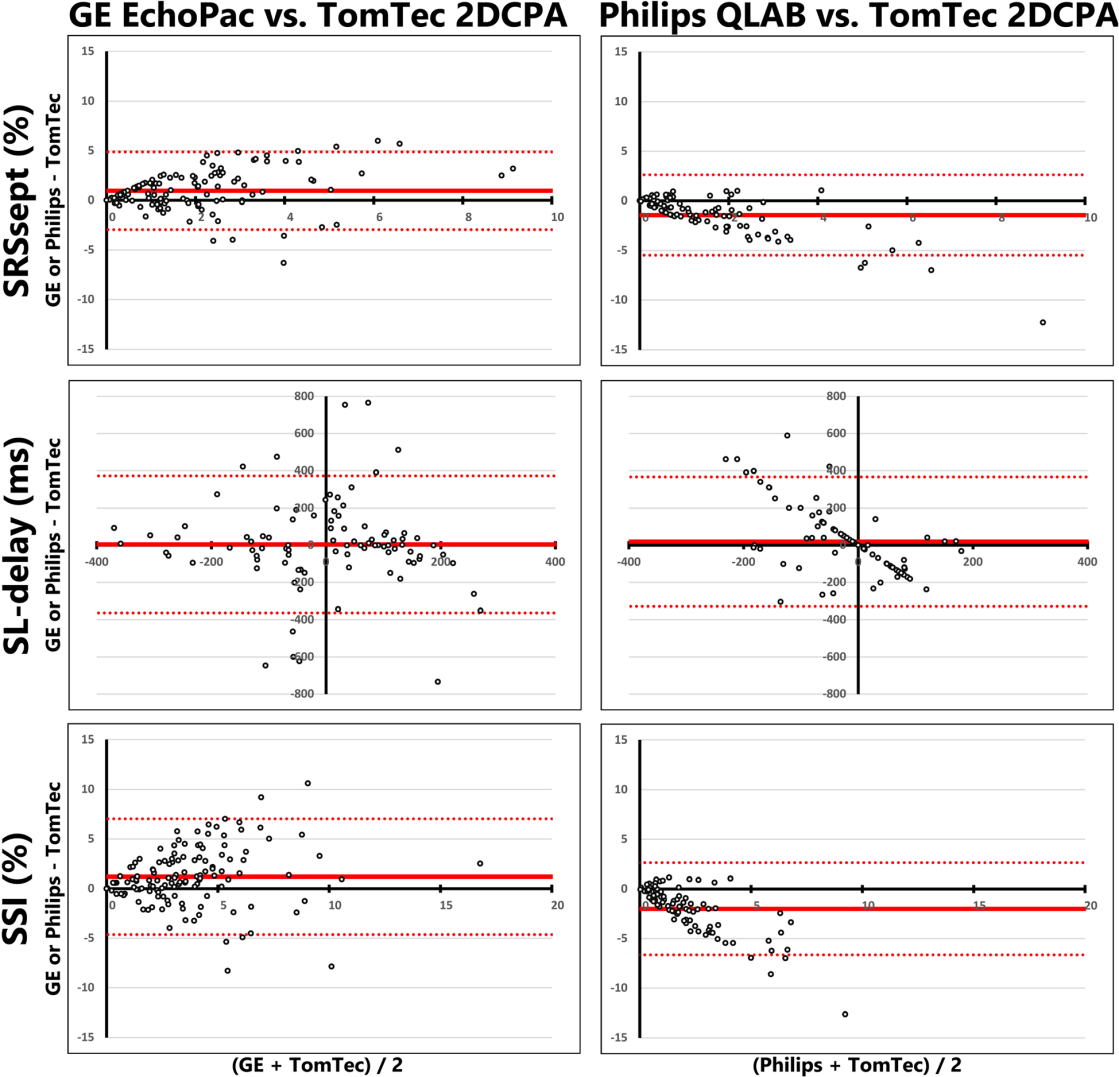
Bland-Altman plots of dyssynchrony parameters, comparing GE Echopac and TomTec 2DCPA (left panels) and Philips QLAB and TomTec 2DCPA (right panels derived results. On each x-axis the average result of the two techniques is given per patient, while on the y-axis the difference is given., GE: General Electric, SRSsept: systolic rebound stretch of the septum, SL-delay: septal to lateral wall delay, SSI: systolic stretch index

**Fig. 5:**
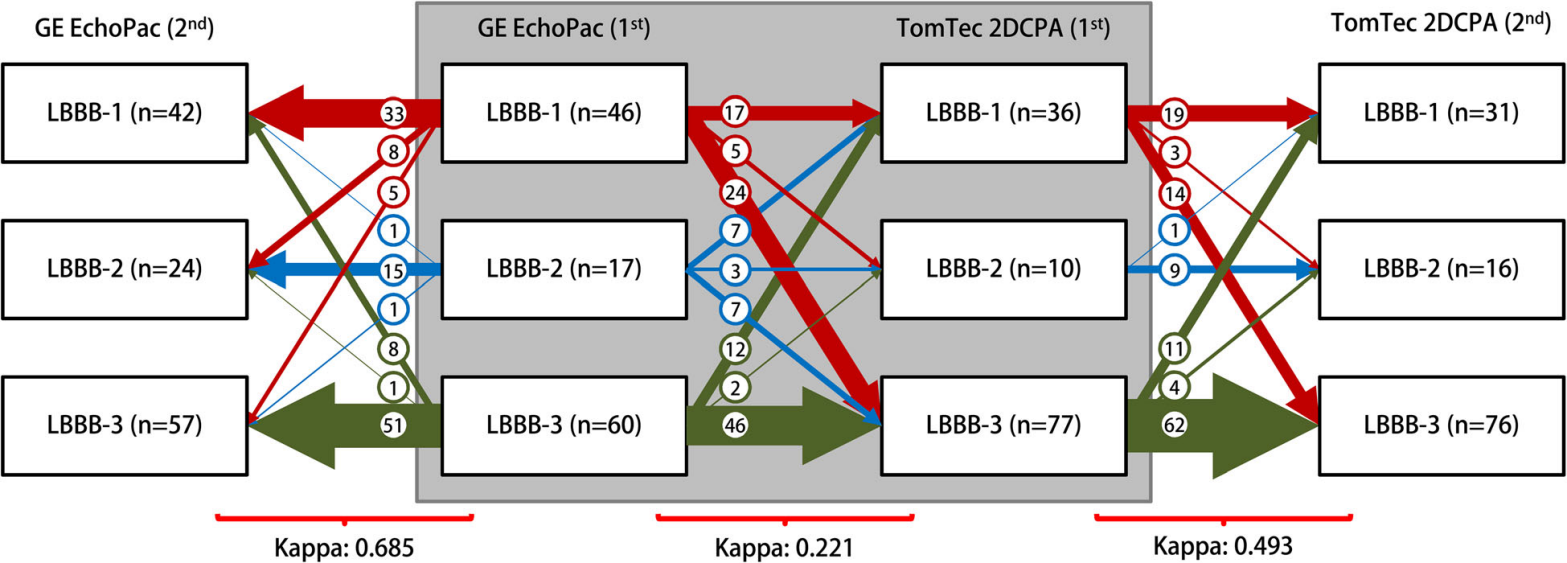
Schematic overview of septal strain pattern categorization for GE derived echocardiographic images, analysed with GE EchoPac and TomTec 2DCPA. Agreement between vendor-specific (GE EchoPac and vendor-independent (TomTec 2DCPA) software is given in the grey square, with corresponding Cohen’s kappa given underneath. Both analyses were performed twice (1st and 2nd), to determine the intra-observer agreement. Arrows indicate the reclassification of patients between vendors or between the first (1st) and second attempt (2nd). LBBB-1: double-peaked systolic stretch, LBBB-2: predominant stretch, and LBBB-3: pseudo normal shortening

Dyssynchrony indices derived from GE images showed varied results. SL-delay showed a poor ICC (0.078) and high COV (−14.4) and wide limits of agreement in the Bland-Altman plots (mean difference: 2 ± 226 ms). The ICC of SRSsept was weak (0.470), COV was relatively high (0.937) and the Bland-Altman plots showed relative wide limits of agreement (1.0 ± 2.0%, Fig. 4). SSI showed similar results, ICC was also weak (0.467), COV was relatively high (0.720) and Bland-Altman plots showed a difference between vendors with relative wide limits of agreement (1.3 ± 3.0%, Fig. 4). Cohen’s kappa coefficient of agreement on LBBB pattern categorization was low (0.221). Cohen’s kappa coefficient of intra-observer agreement was good for GE EchoPac (0.685) and weak for TomTec 2DCPA analysis (0.493) (Fig. 5).

### Philips echocardiographic images

#### Philips basic strain parameters

**Fig. 6:**
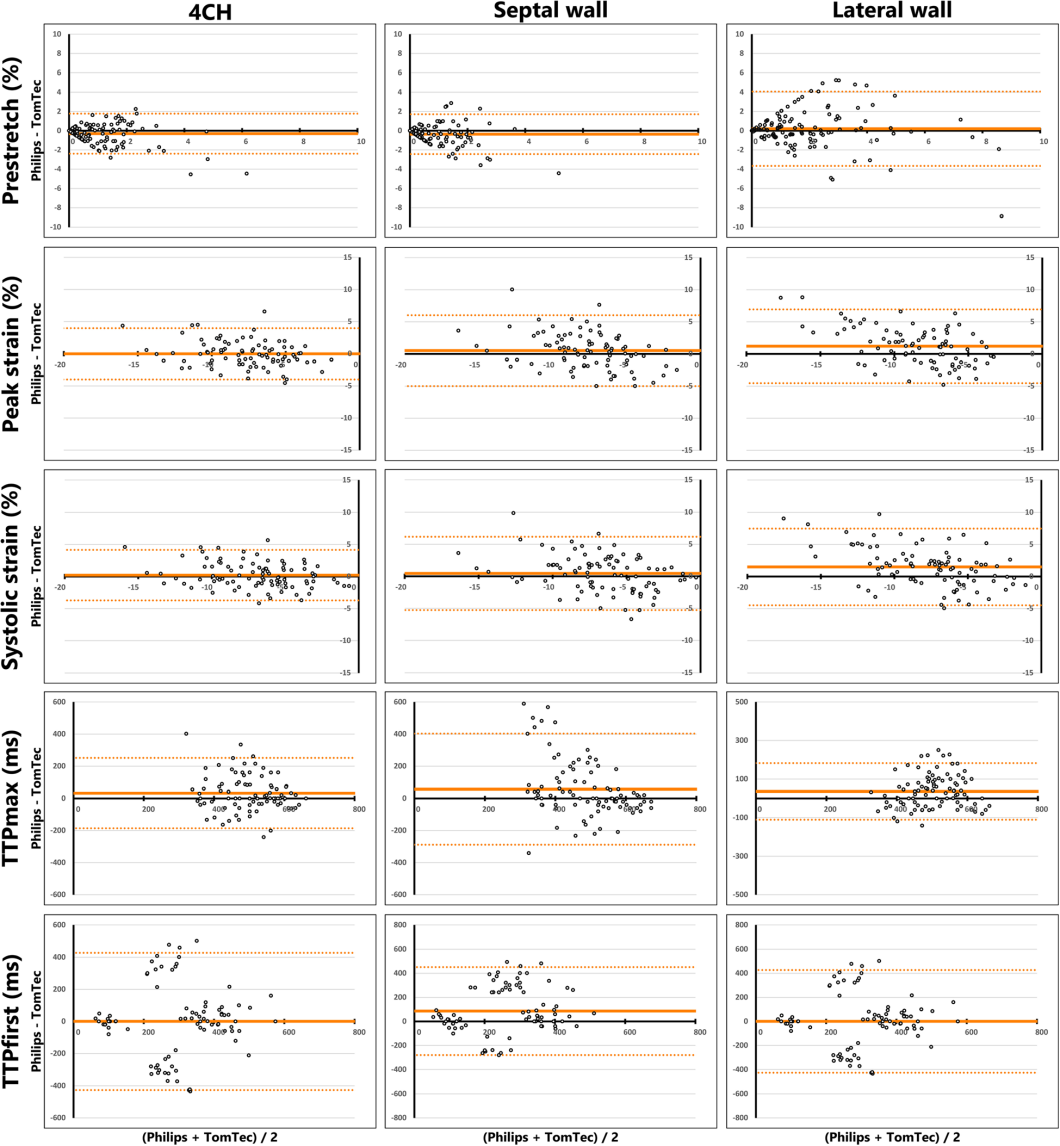
Bland-Altman plots of all strain parameters, comparing Philips QLAB and TomTec 2DCPA derived results. Each column represents either results obtained from the total LV or from the separate septal or lateral wall. On each x-axis the average result of the two techniques is given per patient, while on the y-axis the difference is given. 4CH: apical four chamber view, TTP_first_: time to first peak, TTP_max_: time to maximal peak

Comparison of vendor dependent and independent STE results derived from Philips echocardiographic images showed a similar pattern in results to GE (Table 3). Namely, peak and systolic strain showed a smaller bias and COV than pre-stretch, timing parameters (i.e. TTP_max_ and TTP_first_) and dyssynchrony indices. ICCs were overall lower than GE derived results. Peak strain and systolic strain of the global LV showed an excellent ICC (0.749 and 0.802 respectively), with a relatively low COV and low mean difference in Bland-Altman plots (Table 3 and Fig. 6). The ICC of peak and systolic strain of the septal and lateral wall were good (ranging from 0.626 to 0.680). Results on pre-stretch showed a high COV, poor ICC and wide limits of agreement in Bland-Altman results for all three comparisons (i.e. global LV, septal and lateral wall).

**Table 3:** Strain parameters derived from Philips echocardiographic images

	Philips QLAB (*n* = 88)	TomTec 2DCPA (*n* = 88)	COV	ICC (*p*-value)	Bland-Altman (mean diff ±SD)
LV					
1) Pre-stretch (%)	0.0 (0.0–0.0)	0.0 (0.0–0.2)	4.125	−0.052 (0.684)	−0.1 ± 0.6
2) Peak strain (%)	−7.7 ± 2.7	−7.7 ± 3.3	−0.350	0.749 (<0.001)	0.0 ± 2.1
3) Systolic strain (%)	−6.8 ± 3.0	−7.0 ± 3.5	−0.435	0.802 (<0.001)	0.2 ± 2.0
4) TTP_max_ (ms)	527 (444–592)	492 (396–559)	0.185	0.376 (<0.001)	34 ± 113
5) TTP_first_ (ms)	361 (112–438)	361 (118–413)	0.567	0.165 (0.061)	2 ± 213
Septum					
1) Pre-stretch (%)	0.0 (0.0–0.0)	0.0 (0.0–0.4)	3.990	−0.035 (0.627)	−0.3 ± 0.9
2) Peak strain (%)	−7.6 ± 2.7	−8.0 ± 3.7	−0.363	0.626 (<0.001)	0.5 ± 2.8
3) Systolic strain (%)	−6.5 ± 3.0	−7.0 ± 3.9	−0.468	0.667 (<0.001)	0.5 ± 2.9
4) TTP_max_ (ms)	541 (442–598)	477 (346–575)	0.202	0.109 (0.155)	61 ± 179
5) TTP_first_ (ms)	361 (118–426)	161 (114–342)	0.534	0.232 (0.014)	84 ± 185
Lateral wall					
1) Pre-stretch (%)	0.0 (0.0–0.1)	0.3 (0.0–0.8)	2.728	0.324 (<0.001)	−0.5 ± 1.0
2) Peak strain (%)	−8.0 (−9.4 - -6.1)	−9.0 (−11.1 - -6.3)	−0.345	0.631 (<0.001)	1.3 ± 3.0
3) Systolic strain (%)	−6.4 (−9.0 - -5.2)	−8.0 (−11.1 - -4.9)	−0.436	0.680 (<0.001)	1.5 ± 3.0
4) TTP_max_ (ms)	542 (454–597)	476 (434–538)	0.175	0.531 (<0.001)	36 ± 87
5) TTP_first_ (ms)	376 (107–461)	433 (216–481)	0.550	0.060 (0.290)	−41 ± 239
Dyssynchrony					
a) SL-delay (ms)	0 (0–0)	−20 (−121–120)	−10.694	0.025 (0.409)	24 ± 180
b) SRSsept (%)	0.7 (0.3–1.2)	1.7 (0.6–3.3)	1.030	0.419 (<0.001)	−1.5 ± 2.1
c) SSI (%)	0.8 (0.4–1.5)	2.3 (1.1–4.2)	1.024	0.421 (<0.001)	−2.0 ± 2.4
d) LBBB type (n, %)					
LBBB-1	33 (38.4%)	33 (37.5%)			
LBBB-2	3 (3.5%)	11 (12.5%)			
LBBB-3	50 (58.1%)	44 (50.0%)			

Means and standard deviations are given with ± symbol, for non-normal distributed data, the median is given with the interquartile range between brackets

*COV* coefficient of variation, *ICC* intra-class correlation coefficient, diff: difference, *SD* standard deviation, *LV* strain derived from global LV in apical four chamber view, *TTP*_*max*_ time to maximal peak shortening, *TTP*_*first*_ time to first peak shortening, *SL-delay* time delay between septal and lateral peak shortening, *SSI* systolic stretch index, *SRSsept* septal systolic rebound stretch, *LBBB type* type of LBBB strain patterns, based on definition by Leenders et al. [23]

#### Philips dyssynchrony parameters

**Fig. 7:**
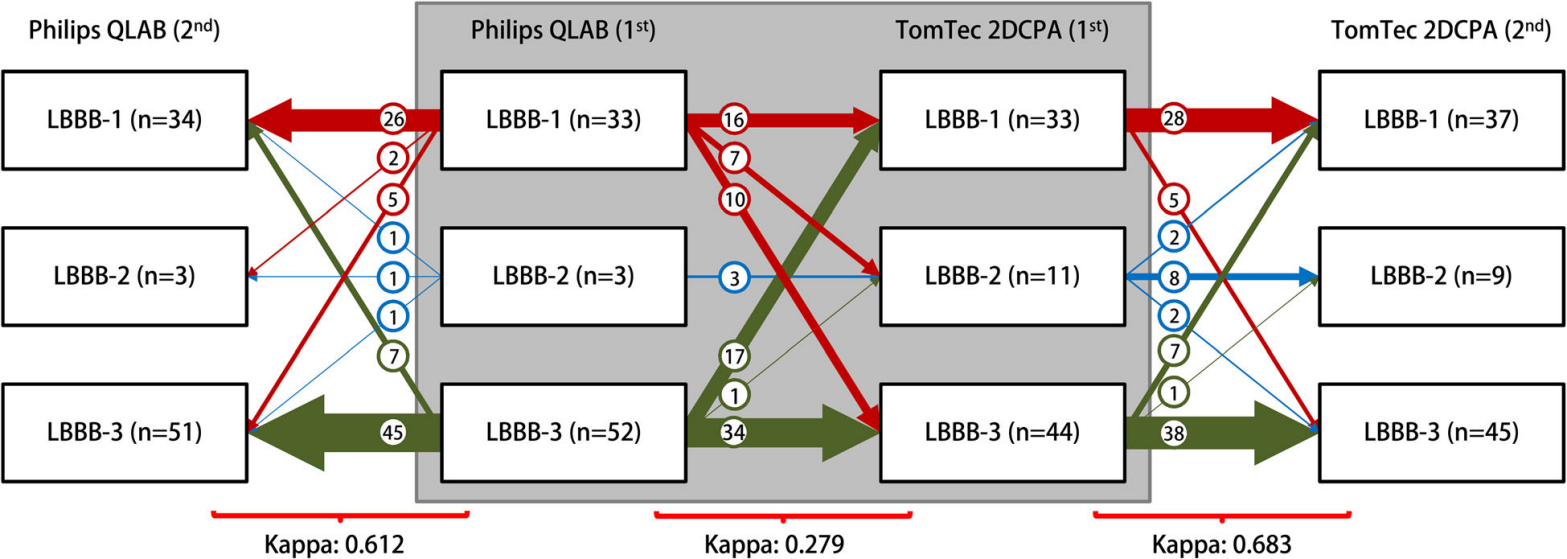
Schematic overview of septal strain pattern categorization for Philips derived echocardiographic images, analysed with Philips QLAB and TomTec 2DCPA. Agreement between vendor-specific (Philips QLAB) and vendor-independent (TomTec 2DCPA) software is given in the grey square, with corresponding Cohen’s kappa given underneath. Both analyses were performed twice (1st and 2nd), to determine the intra-observer agreement. Arrows indicate the reclassification of patients between vendors or between the first (1st) and second attempt (2nd). LBBB-1: double-peaked systolic stretch, LBBB-2: predominant stretch, and LBBB-3: pseudo normal shortening

For Philips vs. TomTec, results on comparison of dyssynchrony parameters were lower for SL-delay (ICC: 0.025, COV: -10.7, Bland-Altman mean difference: 24 ± 180 ms) compared to SRSsept (ICC: 0.419, COV: 1.03, Bland-Altman mean difference: −1.5 ± 2.1%, Fig. 4) and SSI (ICC: 0.421, COV: 1.024, Bland-Altman mean difference: −2.0 ± 2.4%, Fig. 4). Cohen’s kappa coefficient of agreement on LBBB pattern categorization was poor (0.279). The Cohen’s kappa coefficient of intra-observer agreement was good for both QLAB (0.612) and TomTec 2DCPA analysis (0.683) (Fig. 7).

### Cross-correlation

Septal wall cross correlation was significantly lower compared to the LV and lateral wall for GE vs. TomTec (septum: 0.682 ± 0.290, LV: 0.835 ± 0.213, lateral wall: 0.800 ± 0.244, *p* < 0.05) and for Philips vs. TomTec ((septum: 0.712 ± 0.293, global LV: 0.898 ± 0.156, lateral wall: 0.827 ± 0.226, *p* < 0.05). There was no apparent statistical difference between the three subgroups based on image quality (Additional file 2: Table S1). Only for the lateral wall in GE vs. TomTec did the high-quality images (0.892 ± 0.123) have significantly higher R^2^ compared to the poor-quality images (0.713 ± 0.312, *p* < 0.05).

### Prediction of volumetric response

#### GE echocardiographic images

For GE derived images, GE EchoPac derived SRSsept, SSI, and LBBB pattern categorization showed a significant association with volumetric response to CRT in univariate analysis, while TomTec 2DCPA derived parameters did not (Table 4). The SL-delay showed no significant association with volumetric response. C-statistic values were comparable between GE EchoPac and TomTec 2DCPA Except for SSI, cut-off values for response prediction were higher for GE EchoPac (SL-delay: 144 ms, SRSsept: 1.61% and SSI: 2.98%) compared to TomTec 2DCPA (SL-delay: -101 ms, SRSsept 0.46%, SSI: 3.72%).

**Table 4:** Prediction of volumetric response to CRT with GE derived echocardiographic images

	Univariate analysis (*n* = 123)			Receiver operating characteristics (*n* = 123)	
Parameter	B	SD	*p*-value	C-statistic	Cut-off value
*GE* SL-delay	0.820	12.997	0.950	0.512	0.144
*TomTec* SL-delay	22.334	13.081	0.091	0.573	−0.101
*GE* SRSsept	3.146	0.911	<0.001	0.599	1.614
*TomTec* SRSsept	0.503	1.362	0.713	0.544	0.455
*GE* SSI	2.296	0.653	<0.001	0.619	2.980
*TomTec* SSI	1.250	0.818	0.129	0.530	3.715
*GE* LBBB-type (type 1 or 2 vs. 3)	18.536	4.003	<0.001		
*TomTec* LBBB-type (type 1 or 2 vs. 3)	8.151	4.464	0.071		

Prediction of volumetric response to CRT, with results of univariate regression analysis (B, SD and *p*-value) and receiver operating characteristics (C-statistic, R^2^ and cut-off). Univariate analyses are based on a change in LVESV on a continuous scale, while receiver operating characteristics are based on a cut-off of ≥15% reduction in LVESV

*GE* General Electric EchoPac, *TomTec* TomTec 2DCPA, *B* beta coefficient, *SD* standard deviation, *SL*-*delay* septal-to-lateral wall delay, *SRSsept* septal systolic rebound stretch, *SSI* systolic stretch index, *LBBB*-*type* septal strain pattern categorization according to Leenders et al

#### Philips echocardiographic images

For Philips derived images, both Philips QLAB and TomTec 2DCPA showed a significant association with volumetric response to CRT for SRSsept, SSI and LBBB pattern categorization (Table 5). Only the SL-delay showed no significant association with volumetric response. The C-statistic values were overall reasonable (i.e. ranging from 0.564 to 0.705) and comparable between vendor dependent and independent analysis. The cut-off values for response prediction were apparently different, with lower values for Philips QLAB (SL-delay: 0 ms, SRSsept: 0.79% and SSI: 0.83%) compared to TomTec 2DCPA (SL-delay: -80 ms, SRSsept: 1.18% and SSI: 2.35%).

**Table 5:** Prediction of volumetric response to CRT with Philips derived echocardiographic images

	Univariate analysis (*n* = 88)			Receiver operating characteristics (*n* = 88)	
Parameter	B	SD	*p*-value	C-statistic	Cut-off value
*Philips* SL-delay	58.897	38.368	0.129	0.564	0.000
*TomTec* SL-delay	−23.910	16.952	0.162	0.569	−0.080
*Philips* SRSsept	10.072	2.653	<0.001	0.697	0.790
*TomTec* SRSsept	3.842	0.997	<0.001	0.686	1.180
*Philips* SSI	7.346	2.257	0.002	0.661	0.830
*TomTec* SSI	3.860	0.863	<0.001	0.705	2.345
*Philips* LBBB-type (type 1 or 2 vs. 3)	20.091	5.418	<0.001		
*TomTec* LBBB-type (type 1 or 2 vs. 3)	22.069	5.122	<0.001		

Prediction of volumetric response to CRT, with results of univariate regression analysis (B, SD and *p*-value) and receiver operating characteristics (C-statistic, R^2^ and cut-off). Univariate analyses are based on a change in LVESV on a continuous scale, while receiver operating characteristics are based on a cut-off of ≥15% reduction in LVESV

*Philips* Philips QLAB, *TomTec* TomTec 2DCPA, *B* beta coefficient, *SD* standard deviation, *SL*-*delay* septal-to-lateral wall delay, *SRSsept* septal systolic rebound stretch, *SSI* systolic stretch index, *LBBB*-*type* septal strain pattern categorization according to Leenders et al

## Discussion

Comparability of speckle tracking echocardiography platforms on apical four chamber LV peak and systolic strain is fair in patients with heart failure and dyssynchrony. We observed relevant differences in more specific strain parameters (i.e. pre-stretch, TTP_max_ and TTP_first_) and indices representing dyssynchrony (i.e. SRSsept, SSI and SL-delay). Results on strain pattern categorization (i.e. LBBB patterns) were disappointing as agreement between vendors was low. However, the inter-observer agreement, using the same STE software twice, on strain pattern categorization was better. Although most dyssynchrony parameters showed a weak but significant association with changes in LV end-systolic volume, the cut-off values were apparently different. STE software of different vendors can therefore not be used interchangeably for more specific purposes than peak strain.

### Vendor variability

To the best of our knowledge, this is the first study to compare results of different STE software packages, specifically for mechanical dyssynchrony in CRT-candidates. The average differences of peak strain and systolic strain were small and non-significant between vendor dependent and vendor independent STE packages. Nevertheless, TomTec had lower values compared to GE EchoPac and higher values compared to Philips QLAB. Unfortunately, we cannot define the source of discordance, as a gold-standard for deformation imaging (i.e. sonomicrometry) was not available in our study. The relative high correlation for global longitudinal strain between STE platforms is in accordance with earlier publications [16]. There are currently no publications on vendor comparison studies on STE in patients with heart failure and dyssynchrony, besides a small comparative study by our own group [24]. Moreover, comparison to earlier publications on peak strain and timing values is difficult as previous studies implemented older versions of STE software, while we used the most recent versions. STE software is constantly under development, partly due to the STE standardization taskforce of the EACVI/ASE. This task force includes among its members representatives of several vendors. Their efforts resulted in small and acceptable differences between vendors for global longitudinal strain [16, 25]. However, the variability among vendors in more specific longitudinal strain features is not yet elucidated, nor is the exact bias between vendors with respect to regional strain assessment. Furthermore, the cohort studied for standardization consisted of a wide range of subjects (mean LVEF 60%, global longitudinal strain −19.2%) and is therefore not representative for CRT patients with dilated hearts, reduced LV function, and complex deformation characteristics [23]. Moreover, CRT patients can have suboptimal acoustic windows which affects image quality and reliability of strain analysis. In the current study, comparable 4CH peak longitudinal strain values were found in CRT patients, although the limits of agreement of Bland-Altman plots were relatively wide, and results for individual patients varied significantly. The discrepancies between the current study and the publications by Farsalinos et al.. and Yang et al. may therefore be ascribed to the examined populations [16, 26]. A mechanistic modelling study showed higher variability in peak strain among vendors and a higher inter-observer variability in a dilated thin-walled LV [27]. This modelling study suggests a lower level of agreement among vendors in heart failure patients, which might explain the findings in our current observations.

### Echocardiographic images and speckle tracking algorithms

Differences between manufacturers are largely attributed to discrepancies in STE algorithms. Albeit recently thoroughly investigated, [16] the algorithms of the majority of commercially available speckle tracking software have lacked published validation [27]. They are furthermore not open-source. TomTec 2DCPA uses DICOM images and thereby imports images with lower frame rate and lower image quality compared to the raw image files used by the vendor dependent platforms. Lower frame rates influence temporal resolution, which hampers reliable assessment of both strain values and timing indices. The image quality directly influences spatial resolution, decreasing reliable tracking of speckles. TomTec also displays separate endo- and epicardial strain curves for each segment, and mean myocardial wall strain results are not given. The use of endocardial strain data might have caused a slight overestimation of peak strain values [22]. GE EchoPac uses ‘global’ wall myocardial strain by default, although users can choose between endocardial, epicardial or mid myocardial layers. Lastly, the method used by Philips QLAB is unknown, although a global myocardial based approach is likely. Timing of the reference length is also of importance for standardization, as differences in the onset of strain curves directly influences absolute strain values as wells as timing indices. As mentioned, timing of reference length was uniformed for TomTec analysis compared to both vendor dependent platforms.

### Mechanical dyssynchrony indices

Absolute values of mechanical dyssynchrony indices were significantly lower for Philips, compared to TomTec. Whereas the results on dyssynchrony parameters obtained from GE images displayed higher values for GE compared to TomTec. Although the source of discordance is unknown, dyssynchrony seems underestimated by Philips QLAB speckle tracking algorithms. Underestimation of dyssynchrony is exemplified by results for the SL-delay obtained by Philips QLAB (median 0 ms, interquartile range 0 – 0 ms). The discrepancies of Philips QLAB with both other vendors are remarkable, as both GE EchoPac and TomTec 2DCPA displayed large variation for SL-delay. Moreover, as the Bland-Altman plot of SL-delay in Fig. 4 shows, the discrepancy between Philips QLAB and TomTec 2DCPA, a large number of results are on a line (y = −0.5*x), indicating a large variation in SL-delay for TomTec, while Philips values were mainly close to zero. Philips’ derived septal and lateral wall strain curves were often quite similar, as can be appreciated in the example in Fig. 1. It seems that segmental strain curves are more smoothened by Philips QLAB. While no gold-standard for deformation imaging was applied, the relative absence of dyssynchrony obtained with Philips STE software is striking.

Although intra-observer agreement of strain pattern categorization is relatively good, strain pattern categorization showed apparent variations among vendors. Strain patterns were earlier found to be more robust between vendors [24]. This discrepancy could be attributed to changes in STE algorithms, as there were almost no LBBB type 2 patterns found by Philips. LBBB type 2 is the most distinctive septal deformation pattern, with predominant stretch almost in completely opposite direction to the lateral wall. Higher percentages of LBBB type 2 were observed in the same (i.e. Philips imaged) patient with TomTec. The cohorts of GE and Philips were not significantly different, and conventional dyssynchrony parameters such as apical rocking, septal flash and IVMD were comparable. Therefore, the relative absence of LBBB type 2 patterns is likely caused by the inability to detect dyssynchrony using QLAB. Given the above-mentioned differences in both continuous and categorical dyssynchrony parameters, one might postulate that STE with Philips QLAB is less suitable for detection of dyssynchrony in a CRT population. However, despite the lower values, the predictive value of Philips QLAB derived dyssynchrony parameters is at least comparable to the vendor independent analysis of TomTec 2DCPA. Although Philips QLAB and TomTec 2DCPA were able to predict volumetric response to CRT with the implemented dyssynchrony parameter, the cut-off values were different. Even though cut-off values for GE EchoPac derived parameters were higher, the values are different from earlier published values [3, 9]. These differences may be ascribed to the used software versions or the examined populations. Vendor specific cut-off values should therefore be used for each STE platform.

### Echocardiographic image quality

Echocardiographic analysis in the current study was restricted to 4CH images, as speckle tracking analysis of these images is relevant for dyssynchrony in patients with left bundle branch block and has higher reproducibility [15]. However, echocardiographic 4CH images with adequate image quality can be difficult in patients with dilated hearts. Patient anatomy and cardiac size both complicate echocardiographic acquisition, as can be observed from the number of echocardiograms with poor image quality. Although this is not reflected in our results, lateral wall acquisition can be difficult in heart failure patients. It was surprising that lateral wall cross-correlation values were significantly higher compared to septal wall values for all vendors. Lower septal wall cross-correlation values can be explained by the higher complexity of septal strain patterns (i.e. LBBB type 1 and 2, Fig. 2). This in contrast to strain patterns of the lateral wall, which often had a similar shape between patients, also seen in the agreement on TTP_max_ for lateral wall strain. Complex septal deformation pattern can more easily be misinterpreted, resulting in lower correlations and wider limits of agreement in Bland-Altman plots. The poor agreement in septal strain analysis was also seen in the low Cohen’s kappa values of septal strain pattern categorization.

### Limitations

Although this study consists of relatively large subgroups, it is a sub-analysis with inherent limitations. However, images were prospectively collected for analysis with STE software. Nonetheless, patients underwent echocardiographic examination by a single vendor, which was assigned dependent of the centre of implantation and therefore non-randomized. Ideally patients would undergo echocardiographic examination by both vendors, making a direct comparison between GE and Philips possible. Moreover, there was no gold-standard used in this study, making it impossible to determine the source of variability. Test-retest variability was not part of the imaging protocol, although consecutive measurements are subject to variation [16]. Nevertheless, the large and comparable subgroups permitted a reliable comparison of vendors, and large differences were seen. This is in contrast to previous studies, in which most echocardiographic dyssynchrony parameters (i.e. SRSsept, SSI, and SL-delay) were tested solely on one vendor (i.e. GE EchoPac).

### Clinical implications

Although global LV peak strain correlates reasonable between vendor systems, the results of individual patients between vendors may vary, as indicated by the wide limits of agreement in Bland-Altman plots. This variation hampers translation of deformation parameters obtained by STE to clinical practice. All three STE vendors were capable to predict response to CRT, using the implemented dyssynchrony parameters. Although the diagnostic value of GE EchoPac derived parameters is well validated in prior studies, [10, 28] further work is needed to confirm the predictive value of these parameters in clinical practice. Differences between vendors can be large, hampering direct translation from pre-clinical work to the clinical implementation of speckle tracking derived dyssynchrony parameters and patterns in all echo laboratories, with a myriad of echo-machines. We recommend that in patients eligible for CRT, clinicians should use reference and cut-off values specific to the STE vendor.

## Conclusions

This study proves the general fair comparability of longitudinal peak strain, although results for individual cases and more complex strain parameters can differ significantly. Moreover, we have demonstrated that dyssynchrony parameters derived with different vendors are associated with volumetric response to CRT, but that cut-off values do not correlate well between vendors. While the standardization taskforce took an important first step for global peak strain, further standardization of STE in patients eligible for CRT is still warranted.

## Additional files

Additional file 1: Figure S1. Study flow diagram. Study flow diagram of the vendor comparison study. A total of 240 patients were included in six medical centres in the Netherlands. For GE EchoPac, 142 patients were included of which 123 echocardiograms were eligible for STE analysis. For Philips QLAB 88 of 98 echocardiograms were eligible for STE analysis. All echocardiograms were also analyzed with TomTec 2DCPA. Potential reasons for exclusions were: technical errors in the data format, low frame rate (<35 Hz), very poor image quality and irregular heart rhythm. MARC: markers of response to cardiac resynchronization therapy. (TIFF 2114 kb)

Additional file 2: Table S1. Cross-correlation of strain curves. (DOCX 17 kb)

## Abbreviations

4CH: Apical four chamber view
ASE: American Society of Echocardiography
COV: Coefficient of variation
CRT: Cardiac resynchronization therapy
EACVI: European Association of Cardiovascular Imaging
ESV: End-systolic volume
GE: General Electric
ICC: Intra-class correlation coefficient
IVMD: Interventricular mechanical dyssynchrony
LBBB: Left bundle branch block
LV: Left ventricular
MARC: Markers and Response to Cardiac Resynchronization Therapy
R^2^: Coefficient of determination
ROI: Region of interest
SL-delay: Septal to lateral wall delay
SRSsept: Systolic rebound stretch of the septum
SSI: Systolic stretch index
STE: Speckle tracking echocardiography
TTP_first_: Time to first peak
TTP_max_: Time to maximal peak
