# Supplementary figures and images for: Comparison of strain parameters in dyssynchronous heart failure between speckle tracking echocardiography vendor systems
Source: Cardiovasc Ultrasound. 2017 Oct 18;15:25. doi: 10.1186/s12947-017-0116-5 (PMC5648447; doi:10.1186/s12947-017-0116-5)

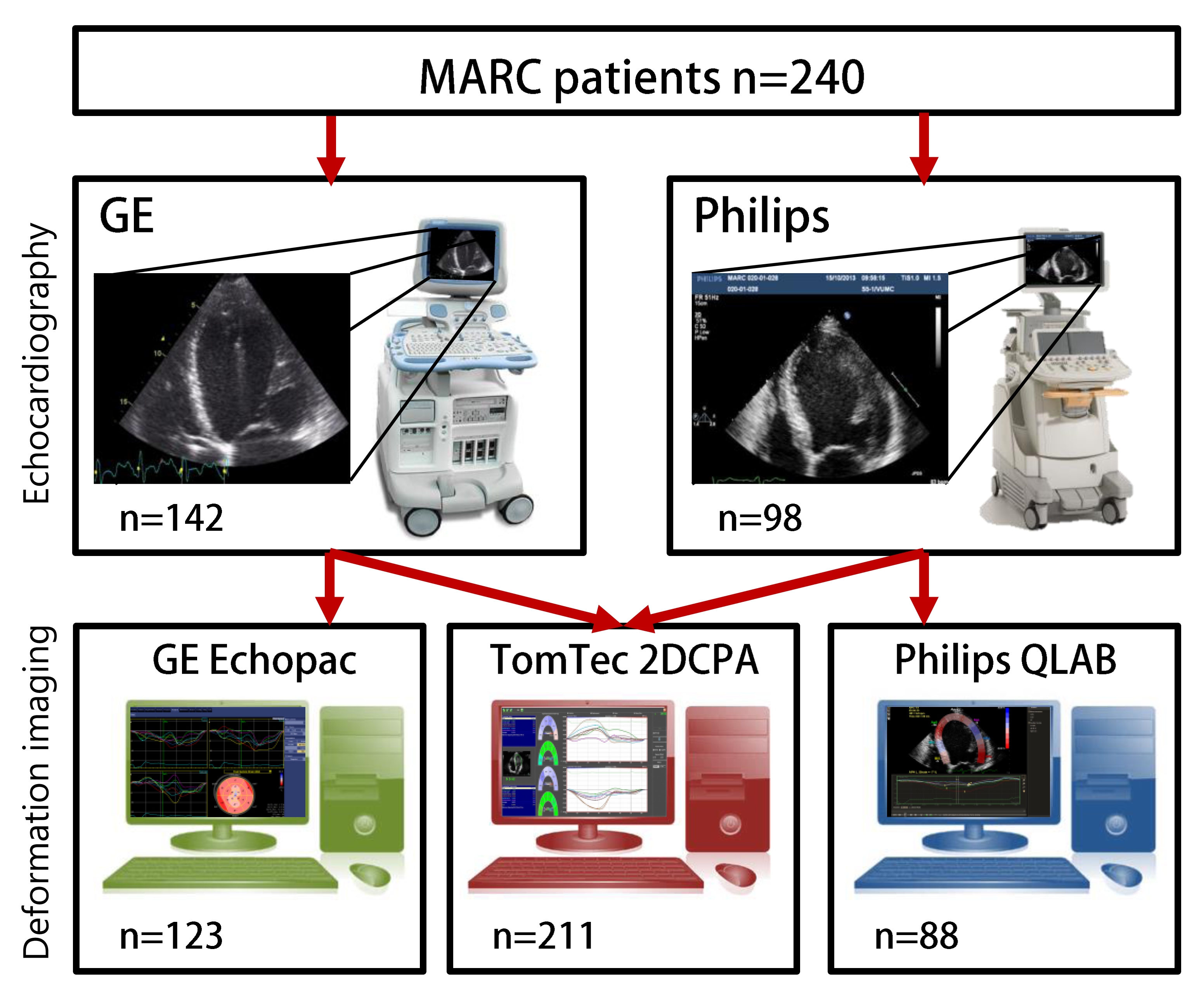

Supplement: Supplementary file 1 — Study flow diagram. Study flow diagram of the vendor comparison study. A total of 240 patients were included in six medical centres in the Netherlands. For GE EchoPac, 142 patients were included of which 123 echocardiograms were eligible for STE analysis. For Philips QLAB 88 of 98 echocardiograms were eligible for STE analysis. All echocardiograms were also analyzed with TomTec 2DCPA. Potential reasons for exclusions were: technical errors in the data format, low frame rate (<35 Hz), very poor image quality and irregular heart rhythm. MARC: markers of response to cardiac resynchronization therapy. (TIFF 2114 kb) [file 12947_2017_116_MOESM1_ESM.tif]
