# Supplementary material for: Comparison of strain parameters in dyssynchronous heart failure between speckle tracking echocardiography vendor systems
Source: Cardiovasc Ultrasound. 2017 Oct 18;15:25. doi: 10.1186/s12947-017-0116-5 (PMC5648447; doi:10.1186/s12947-017-0116-5)
Supplement: Supplementary file 2 — Cross-correlation of strain curves. (DOCX 17 kb) [file 12947_2017_116_MOESM2_ESM.docx]

# Additional file 2

Table S1. Cross-correlation of strain curves

|  | GE EchoPac vs. TomTec 2DCPA | | | |
| --- | --- | --- | --- | --- |
|  | Overall (n=123) | High (n=34) | Average (n=58) | Poor (n=31) |
| Global LV | 0.835±0.213^†^ | 0.903±0.113 | 0.802±0.242 | 0.821±0.226 |
| Septal wall | 0.682±0.290^††^ | 0.729±0.254 | 0.630±0.319 | 0.728±0.257 |
| Lateral wall | 0.800±0.244 ^†^ | 0.892±0.123* | 0.792±0.242 | 0.713±0.312* |
|  |  |  |  |  |
|  | Philips QLAB vs. TomTec 2DCPA | | | |
|  | Overall (n=88) | High (n=30) | Average (n=50) | Poor (n=8) |
| Global LV | 0.898±0.156^††^ | 0.924±0.108 | 0.890±0.154 | 0.850±0.283 |
| Septal wall | 0.712±0.293^††^ | 0.723±0.269 | 0.672±0.319 | 0.905±0.084 |
| Lateral wall | 0.827±0.226^††^ | 0.859±0.201 | 0.829±0.221 | 0.701±0.314 |

Results of cross-correlation of strain curves, categorized by image quality. Mean coefficient of determination (R^2^) and standard deviations are given with ± symbol. Global LV: global 4CH LV results. ^†^: p<0.05 between groups for overall analysis, *: p<0.05 between image qualities.
